# Enhanced cellulase production by decreasing intercellular pH through H^+^-ATPase gene deletion in *Trichoderma reesei* RUT-C30

**DOI:** 10.1186/s13068-019-1536-2

**Published:** 2019-08-13

**Authors:** Pei Liu, Guoxiu Zhang, Yumeng Chen, Jian Zhao, Wei Wang, Dongzhi Wei

**Affiliations:** State Key Lab of Bioreactor Engineering, New World Institute of Biotechnology, East China University of Science and Technology, P.O.B. 311, 130 Meilong Road, Shanghai, 200237 China

**Keywords:** *Trichoderma reesei*, Glucose, H^+^-ATPase, Intercellular pH, Ca^2+^ channels, Calcium signaling

## Abstract

**Background:**

Cellulolytic enzymes produced by *Trichoderma reesei* are widely used for the industrial production of biofuels and chemicals from lignocellulose. We speculated that intracellular pH during the fermentation process can affect cellulase induction.

**Results:**

In this study, two H^+^-ATPase genes, *tre76238* and *tre78757,* were first identified in *T. reesei*. Deletion of *tre76238* and *tre78757* in *T. reesei* RUT-C30 confirmed that *tre76238* has a major function in maintaining intracellular pH, whereas *tre78757* has a minor function. The *tre76238* deletion strain Δ76238 displayed a high level of cellulase production using cellulase-repressive glucose as a sole carbon source, along with intracellular acid accumulation and growth retardation. Our results indicated that intracellular acid accumulation in Δ76238 stimulated a significant increase in the cytosolic Ca^2+^ levels. Ca^2+^ channels were shown to be necessary for cellulase production using glucose as the carbon source in Δ76238. Delayed Δ76238 growth could be reversed by optimizing the medium’s nitrogen sources to produce ammonia for intracellular acid neutralization in the early phase. This may be useful for scale-up of cellulase production using glucose as the carbon source.

**Conclusions:**

This study provides a new perspective for significant alterations in the cellulase expression pattern of *T. reesei* Δ76238, indicating a new mechanism for cellulase regulation under conditions of low intracellular pH.

**Electronic supplementary material:**

The online version of this article (10.1186/s13068-019-1536-2) contains supplementary material, which is available to authorized users.

## Background

As the most abundant and renewable resource in nature, lignocellulosic biomass has been widely used to produce biofuels and bioproducts, which have received increased focus for further research [1]. Biological conversion of lignocellulosic biomass into fermentable sugars by cellulosic enzyme usage is an environment-friendly and promising approach. However, the production cost of a large amount of biomass-degrading enzymes is still a significant challenge for commercial biofuel production [2–4]. The filamentous fungus *Trichoderma reesei* is an important producer for most cellulolytic enzymes used in biomass conversion today [5]. The cellulases produced in *T. reesei* mainly comprise two cellobiohydrolases (CBHI and CBHII), two endoglucanases (EGI and EGII), and *β*-glucosidase I (BGLI) [6, 7].

A complex regulatory network is needed for the accurate regulation of cellulase gene expression [8]. Several transcription factors for cellulase gene expression have been characterized in *T. reesei*, including the main transactivator of cellulase and hemicellulase expression, XYR1 [9], as well as the carbon catabolite repressor, CRE1 [10]. In *T. reesei*, CREI is known to repress the transcription of several cellulase genes such as the CBHI-encoding gene *cbh1* and the main transactivator XYRI [11–14], in the presence of d-glucose. XYR1 is considered the essential activator controlling the major cellulase and hemicellulase gene expression [15]. Many cellulase high-producing *T. reesei* mutants have been obtained by classical mutagenesis for several decades. The moderately overproducing strain QM9414 (ATCC 26921) and the *T. reesei* hyperproducing strain RUT-C30 (ATCC 56765) are the most widely used *T. reesei* strains in academic research [6, 16].

The productivity and efficiency of cellulolytic enzymes produced by *T. reesei* is significantly affected by culture pH [17, 18]. Earlier reports have shown that a lower pH during the fermentation process might favor efficient cellulase production [7, 18], whereas a higher pH is essential for xylanase production [19]. The best cellulase production was obtained at a lower pH (4.0 minimum) by Bailey et al. [19]. Some reports have suggested that optimizing pH in fermentation broth can improve and maintain industrial cellulase production in *T. reesei* [17, 20, 21]. However, studies based on regulating and maintaining pH for cellulase production in *T. reesei* were only done by changing the extracellular pH in fermentation broth [7, 18–21]. Extracellular acids may be transported into the cells, affecting the intracellular pH homeostasis and stimulating the upstream (hemi-) cellulase regulation pathway, though this is unclear [22, 23]. The effect of directly changing intracellular pH on cellulase production in *T. reesei* has also aroused great interest.

Carbon source consumption during *T. reesei* growth is sometimes accompanied by acid production [22]. Aside from the acid in the cultural environment, intracellular pH homeostasis is also achieved by multiple regulation of molecules via pumps and exchangers [24]. The fungal plasma membrane H^+^-ATPase is the primary proton pump that exports cellular protons, using ATP as an energy source [23, 25] and plays a key role in intracellular pH homeostasis [23]. Plasma membrane H^+^-ATPases have been functionally characterized in *Saccharomyces cerevisiae*, *Schizosaccharomyces pombe*, and *Neurospora crassa* [26–28]. The major plasma membrane H^+^-ATPases are encoded by the gene *pma1* in *S. cerevisiae* and *S. pombe*. *pma2*, the isogene of *pma1*, is not required for growth [28]. The *pmal* mutants of *S. cerevisiae* were apparently defective at maintaining internal pH, and some mutants were unable to grow either at a low pH or in the presence of a weak acid [29]. Therefore, deleting H^+^-ATPases is a feasible approach for efficiently reducing the intracellular pH.

In this study, plasma membrane H^+^-ATPase genes *tre76238* and *tre78757*, which have a great effect on intracellular pH homeostasis, were identified in *T. reesei* for the first time. Deletion of the gene *tre76238* in *T. reesei* RUT-C30 resulted in significant cellulase production using glucose as the sole carbon source. We further researched the underlying mechanism of significant alterations in the cellulase expression pattern of Δ76238. The medium was also optimized for accelerating Δ76238 growth in the early phase. These findings provide a new strategy for enhanced cellulase production by regulating intracellular pH homeostasis. Our research indicates a new mechanism for cellulase regulation in low intracellular pH conditions, which warrants further research.

## Results

### H^+^-ATPase isogenes *tre76238* and *tre78757* were identified in *Trichoderma reesei*

Fungal plasma membrane H^+^-ATPases play an important role in intracellular pH homeostasis [23]. Two plasma membrane H^+^-ATPase isogenes, *tre76238* and *tre78757*, were identified by a BLAST search of the genome sequence of *Trichoderma reesei* (https://genome.jgi.doe.gov/Trire_Chr/Trire_Chr.home.html [30] or https://genome.jgi.doe.gov/TrireRUTC30_1/TrireRUTC30_1.home.html [31]) for homologs of the H^+^-ATPase genes *pma1* and *pma2* from *Saccharomyces cerevisiae* [32].

The open reading frame of *tre76238* is 2949 bp (CDS Sequence), encoding a 982-amino acid protein TRE76238 with three conserved domains based on comparison to the yeast Pma1 structure [33]. One is a cation ATPase N-terminus (smart00831: residues 74–134). The second conserved domain is an E1-E2 ATPase domain (pfam00122: residues 142–410). The third conserved domain is a P-type ATPase domain (residues 643–701). The open reading frame of *tre78757* is 2772 bp (CDS Sequence). The putative protein TRE78757 contains 923 amino acids, and also has three conserved domains similar to TRE76238. The structure of TRE76238 and TRE78757 proteins was generated using the Phyre2 server [34]. The highest confidence models were based on the *Neurospora* model [35], and are shown in Additional file 1: Fig. S1.

**Fig. 1:**
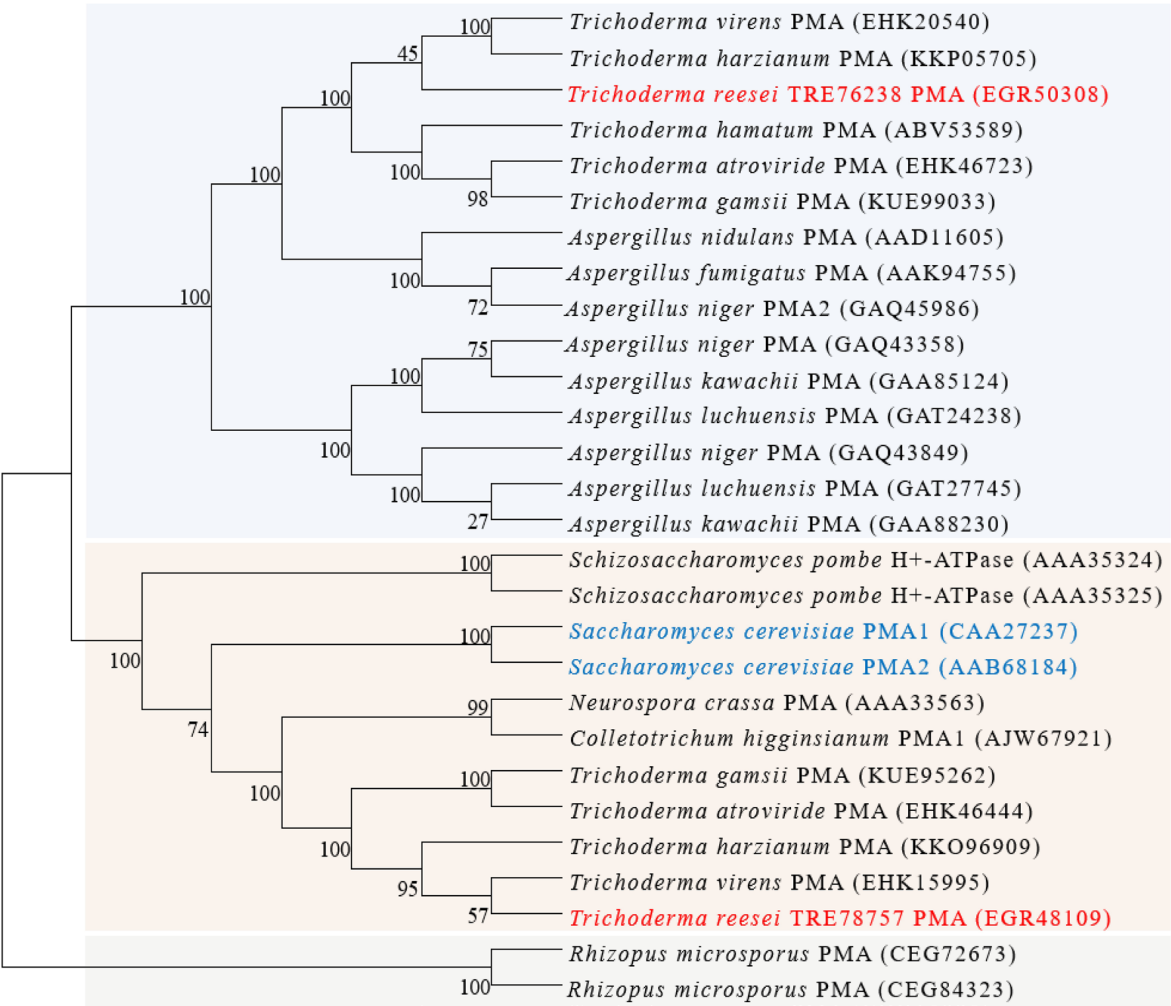
Phylogenetic analysis of TRE76238 and TRE78757. Phylogenetic analysis was performed by MEGA6 using the neighbor-joining method. Bootstrap values are adjacent to each internal node and represent the percentage of 1000 bootstrap replicates. Amino acid sequences were obtained from the NCBI database using the local BLASTp program

Through the phylogenetic analysis shown in Fig. 1, we found that TRE76238 protein and its putative orthologs formed a cluster separate from the TRE78757 orthologs. TRE78757 protein is more closely related to the functionally characterized plasma membrane H^+^-ATPases of *Saccharomyces cerevisiae*, *Neurospora crassa*, and *Schizosaccharomyces pombe* [26]. We predicted that the cluster containing TRE76238 may be specific to some filamentous fungi.

### Deletion of *tre76238* in *T. reesei* RUT-C30 showed growth retardation

**Fig. 2:**
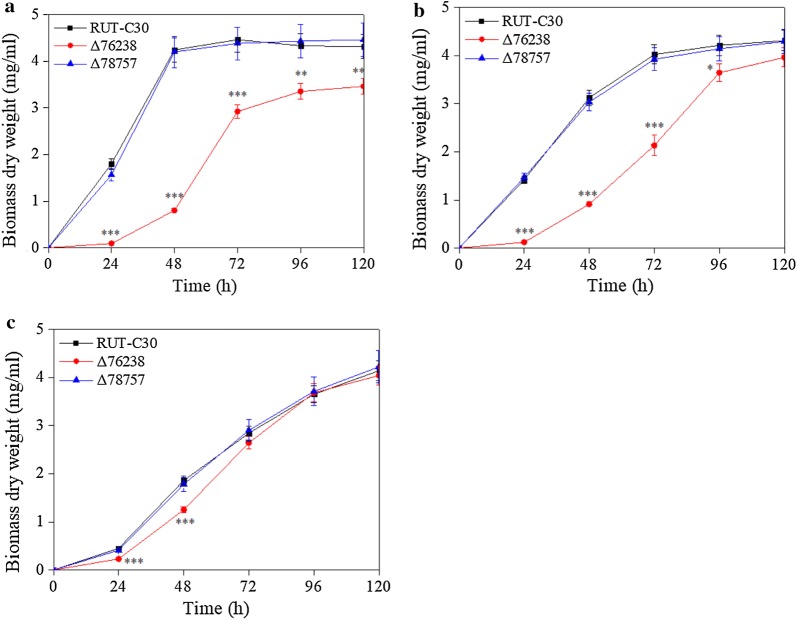
Biomass dry weight of Δ76238, Δ78757, and the parental strain *T. reesei* RUT-C30. The biomass dry weight of *T. reesei* strains was measured in 100 mL MA medium using glucose (**a**), lactose (**b**), and Avicel (**c**) as the sole carbon source. Values are the mean ± SD of results from three independent experiments. Significance was analyzed between the biomass dry weight of *T. reesei* Δ76238 and RUT-C30. Asterisks indicate significant differences (*p < 0.05, **p < 0.01, ***p < 0.001, Student’s *t* test)

This study aimed to examine the effect of reducing intracellular pH on cellulase production by blocking proton transport outside *T. reesei* cells. The genes *tre76238* and *tre78757* were deleted in hyper-cellulolytic *T. reesei* RUT-C30 to form the deletion mutants Δ76238 and Δ78757, respectively. Δ76238 and Δ78757 strains were separately cultivated in 100 mL MA medium with 2% (w/v) Avicel, 2% (w/v) lactose, or 2% (w/v) glucose as the sole carbon source, followed by measurement of the biomass dry weight (Fig. 2).

Knockout of *tre78757* in *T. reesei* RUT-C30 had no significant effect on growth in glucose, lactose, and Avicel (Fig. 2) containing cultures. However, Δ76238 strain growth was significantly decreased (Fig. 2a, b) and formed a mycelium pellet during fermentation when cultured in glucose and lactose (see Additional file 1: Fig. S2). *T. reesei* RUT-C30 biomass accumulation reached a maximum after 48 h of culture. Slow biomass accumulation occurred at the early fermentation phase in Δ76238, which reached its highest level after 96 h using glucose as the carbon source (Fig. 2a). Δ76238 biomass accumulation was also severely delayed using lactose as the carbon source in the early fermentation phase (Fig. 2b). Δ76238 growth in Avicel was similar to that of *T. reesei* RUT-C30, but was only slightly retarded at the early fermentation phase (Fig. 2c). The results indicated that *tre76238* deletion markedly impaired the growth of *T. reesei* RUT-C30 with glucose or lactose as the carbon source; however, this impairment could be partially eliminated using Avicel as the sole carbon source (Fig. 2c).

We tried to delete *tre78757* in the Δ76238 strain to form a double deletion strain. A homokaryotic transformant of the *tre78757* gene could not be obtained upon deleting it in the Δ76238 strain, suggesting that the function of plasma membrane H^+^-ATPase is essential for *T. reesei* RUT-C30. Double deletion of *tre76238* and *tre78757* is lethal for *T. reesei* RUT-C30. The H^+^-ATPase gene *tre76238* is more important for growth than *tre78757* in *T. reesei* RUT-C30.

The most significant difference in Δ76238 growth was found in the glucose condition, followed by lactose, and the least difference was found with Avicel. Therefore, cellulase repressor-glucose and inducer-lactose was used for further research.

### Deletion of gene *tre76238* impairs the ability to transport protons out of cells

To address whether *tre76238* is necessary for intracellular pH homeostasis, the external pH and intracellular pH of the *tre76238* deletion strain Δ76238 was measured using a pH electrode and fluorescent pH probe BCECF-AM (pKa: 6.98, working range is 6.0–8.0), respectively [36]. Using the fluorescent pH probe BCECF-AM, greater green fluorescence intensity of mycelium represented lower proton concentration and higher pH [36]. Green fluorescence was nearly undetected when intracellular pH was less than 6 [37].

**Fig. 3:**
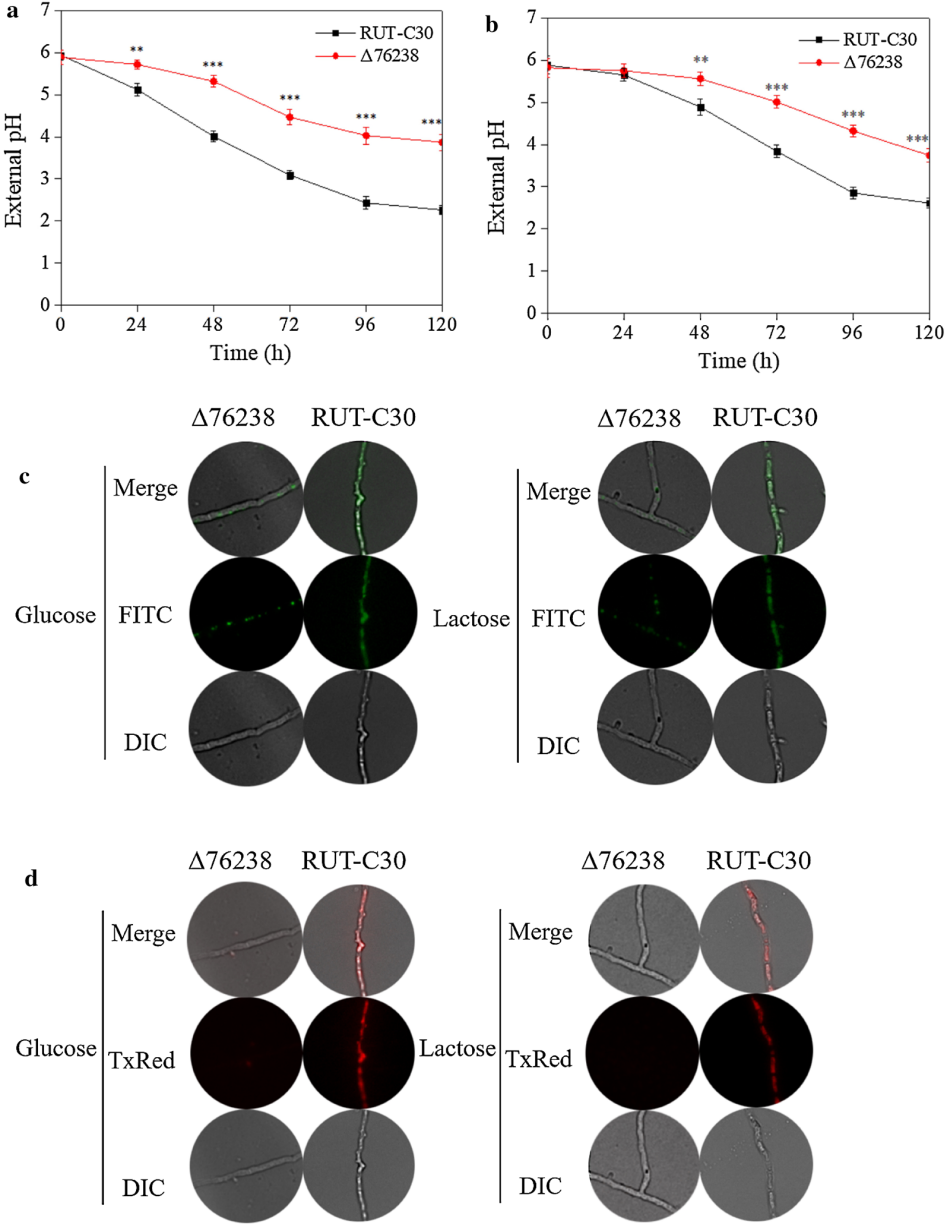
External pH, intracellular pH, and mitochondrial activity of *T. reesei* strains. External pH was measured in Δ76238 and the parental strain *T. reesei* RUT-C30 containing glucose (**a**), and lactose (**b**) as the carbon source. External pH was measured with a pH electrode. **c** Intracellular pH of Δ76238 and parental strain *T. reesei* RUT-C30 was characterized at 96 h, with glucose and lactose as the sole carbon source. Mycelia of *T. reesei* strains were labeled with 5 μM fluorescent pH probe BCECF-AM for 30 min. **d** Mitochondrial activity of Δ76238 and parental strain *T. reesei* RUT-C30 was measured using glucose or lactose as the carbon source at 96 h. Mycelia of *T. reesei* strains were stained with 100 nM MitoTracker^®^ Red CMXRos for 30 min. Values are the mean ± SD of results from three independent experiments. Asterisks indicate significant differences (**p < 0.01, ***p < 0.001, Student’s *t* test)

The external pH of Δ76238 using glucose or lactose as the carbon source is shown in Fig. 3a, b. The external pH of Δ76238 was higher than that of *T. reesei* RUT-C30 using glucose or lactose as the carbon source (Fig. 3a, b). As shown in Fig. 3c for intracellular pH, only a few tiny green fluorescent particles were distributed in the hyphae of the mutant Δ76238 (Fig. 3c) in glucose and lactose conditions, compared with those in the parental strain *T. reesei* RUT-C30. This indicated that most hyphae of Δ76238 were acidic (pH < 6.0), except a few sub-organelles maintaining a pH greater than 6.0. However, whole hyphae of *T. reesei* RUT-C30 exhibited continuous green fluorescence. The intracellular pH of Δ76238 was apparently lower than that of *T. reesei* RUT-C30, showing that proton accumulation occurs in the cytosol of the mutant Δ76238 due to *tre76238* deletion. Therefore, knockout of the H^+^-ATPase gene *tre76238* had a great influence on the intracellular pH in *T. reesei* RUT-C30.

No significant differences in the external and intracellular pH were observed between Δ78757 and parental strains of *T. reesei* RUT-C30 using glucose or lactose as the carbon source (Additional file 1: Fig. S3A–D).

We further characterized the overall biological activity of *T. reesei* Δ76238 by measuring mitochondrial activity. The mitochondria of Δ76238 and *T. reesei* RUT-C30 were characterized by labeling with MitoTracker^®^ Red CMXRos. Red fluorescence was almost undetected in the mutant Δ76238, whereas the hyphae of *T. reesei* RUT-C30 showed marked red fluorescence with glucose as the carbon source (Fig. 3d). No significant differences in mitochondrial activity were observed between Δ78757 and the parental strain *T. reesei* RUT-C30 using glucose or lactose as the carbon source (Additional file 1: Fig. S3E). The results indicated that aerobic metabolism in Δ76238 is very weak. The results also demonstrated that decreased mitochondrial activity was the reason for Δ76238 growth retardation.

Monosaccharide (glucose) and lactose metabolism easily produces acids through glycolysis compared with Avicel [22]. Deleting *tre76238* in *T. reesei* RUT-C30 destroyed the cellular ability to extrude protons from the cells and a large amount of acid produced by glucose metabolism could not be discharged, thus affecting growth. However, as a long-acting carbon source, Avicel was slowly hydrolyzed into glucose, thus accumulating less acid. This could be extruded slowly by TRE78757. Δ76238 growth could be restored by adding an appropriate amount of aqueous ammonia (see Additional file 1: Fig. S4), indicating that the growth delay occurred because of proton accumulation.

### *tre76238* deletion in *T. reesei* RUT-C30 led to high levels of cellulase production using the repressive carbon source glucose

**Fig. 4:**
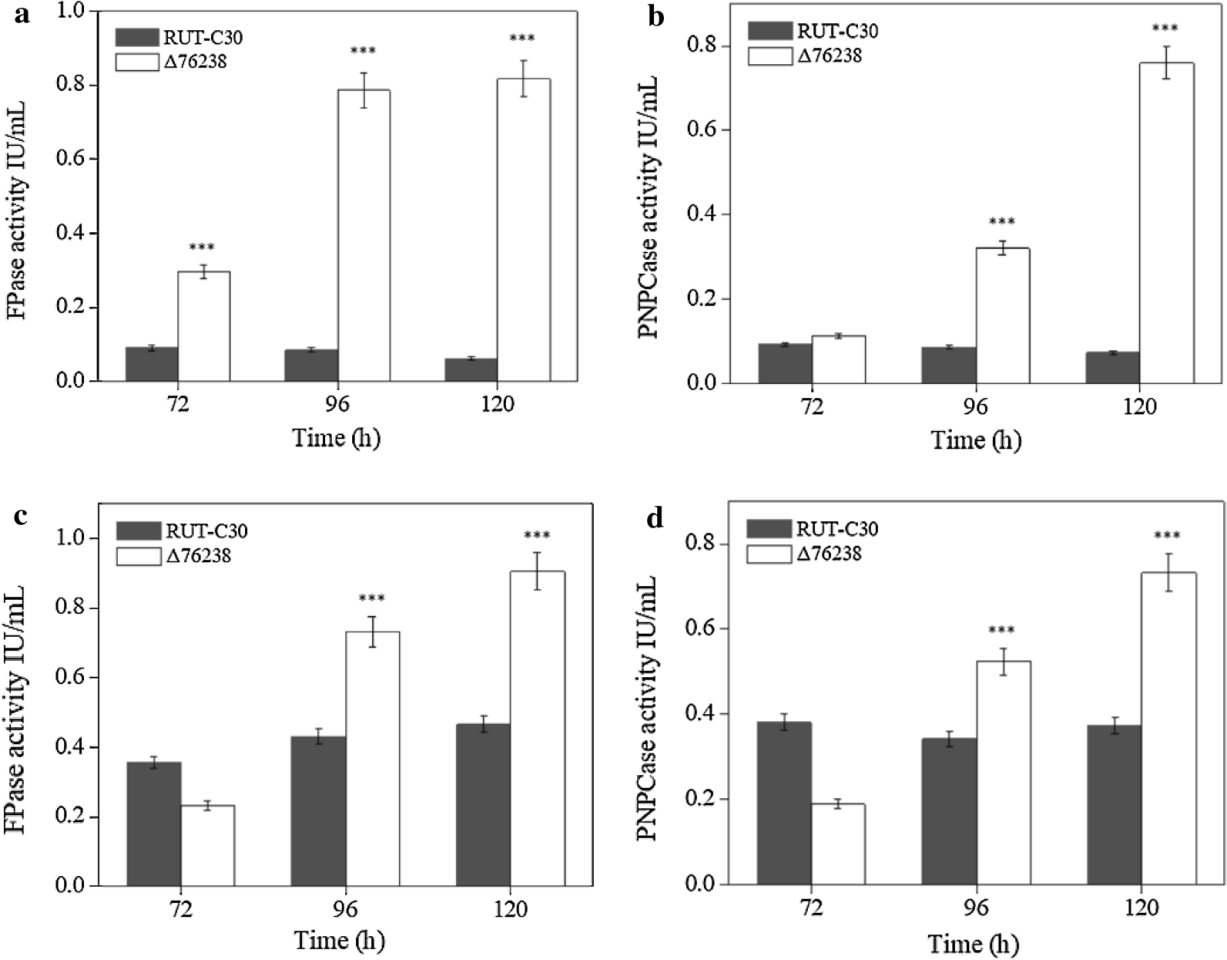
Cellulase production of Δ76238 with glucose and Avicel. The FPase (**a**) and PNPCase (**b**) activities of Δ76238 were measured compared with those of the parental strain *T. reesei* RUT-C30, using glucose as the carbon source. The FPase (**c**) and PNPCase (**d**) activities of Δ76238 were also measured using lactose as the carbon source. Values are the mean ± SD of the results from three independent experiments. Asterisks indicate significant differences (***p < 0.001, Student’s *t* test)

We further measured the cellulase activities of Δ76238, as the intracellular pH of Δ76238 was obviously changed. Surprisingly, Δ76238 exhibited significantly increased cellulase production compared with the parental strain *T. reesei* RUT-C30 using glucose or lactose as the carbon source. As shown in Fig. 4a, b, Δ76238 exhibited high levels of FPase and PNPCase activity (both about 0.8 U/mL) after 5 days of cultivation. Meanwhile, *T. reesei* RUT-C30 had nearly no FPase and PNPCase activity using glucose as the carbon source. Using lactose as the carbon source, Δ76238 demonstrated two times higher FPase and PNPCase activity (both about 0.8 U/mL after 5 days of cultivation) than that of *T. reesei* RUT-C30 (Fig. 4c, d). When Δ76238 was cultivated in Avicel, FPase and PNPCase reached 1.2 U/mL, similar to that of *T. reesei* RUT-C30 (Additional file 1: Fig. S5). It is remarkable that *tre76238* deletion resulted in continuous accumulation of cellulase in *T. reesei* RUT-C30 using glucose as the carbon source.

However, the Δ78757 strain exhibited no significant difference in cellulase production compared with *T. reesei* RUT-C30 in glucose, lactose, and Avicel (Additional file 1: Fig. S6). This was consistent with the lack of growth rate differences, accumulation of intracellular acid, and difference in mitochondrial activity detected between Δ78757 and *T. reesei* RUT-C30.

**Fig. 5:**
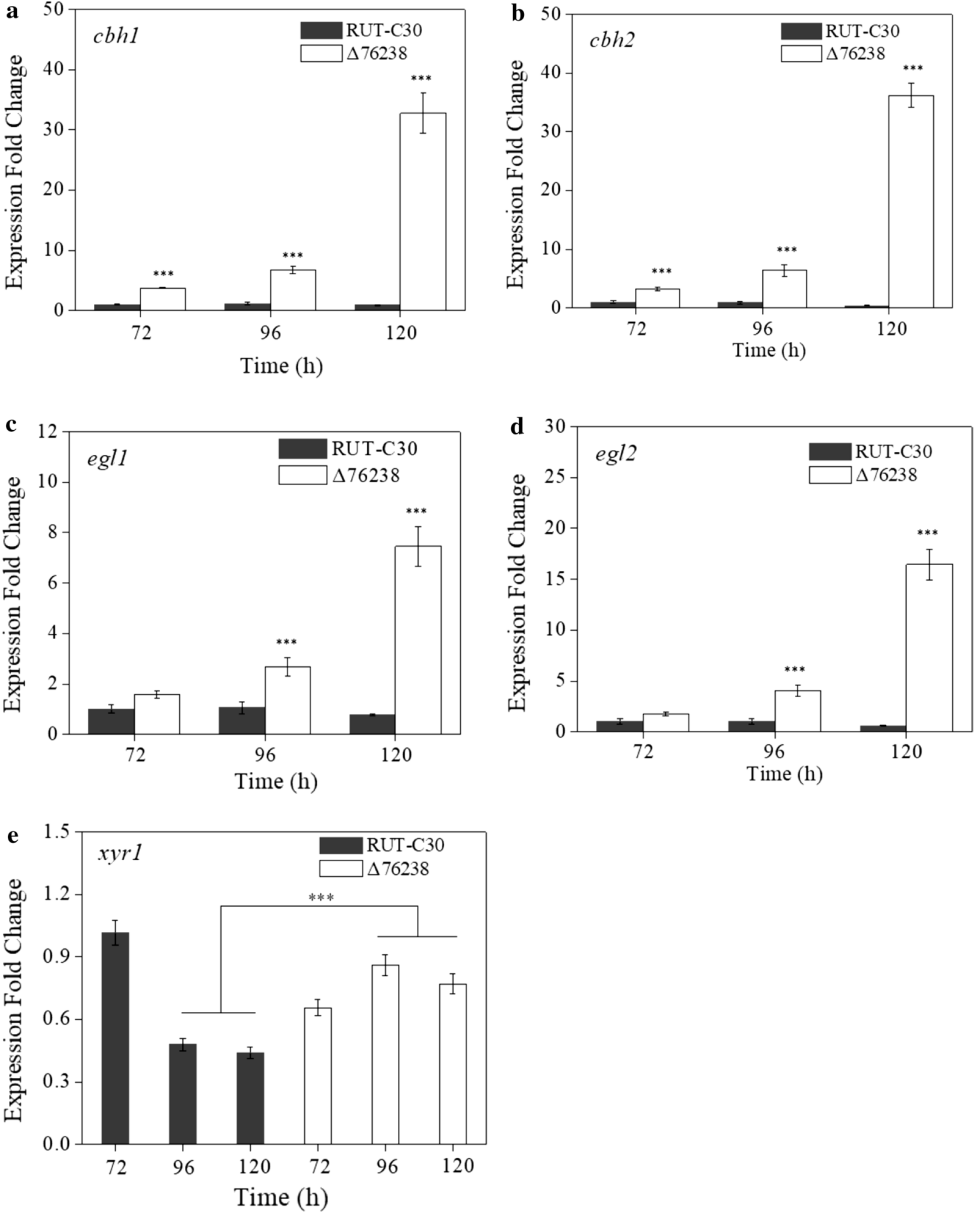
Transcription levels of major cellulase-related genes in Δ76238 using glucose as the carbon source. Transcriptional levels of major cellulase-related genes *cbh1* (**a**), *cbh2* (**b**), *egl1* (**c**), *egl2* (**d**), and *xyr1* (**e**) were evaluated via quantitative real-time PCR (qPCR). *T. reesei* strains were grown using glucose for 72, 96, or 120 h. The data are normalized to the expression of RUT-C30 over 72 h for each tested gene, with the *sar* gene used as an endogenous control in all samples. Values are the mean ± SD of the results from three independent experiments. Asterisks indicate significant differences (***p < 0.001, Student’s *t* test)

To further confirm the effects on enhancing cellulase production, the expression levels of the four main *T. reesei* cellulase genes, *cbh1* (cellobiohydrolase I), *cbh2* (cellobiohydrolase II), *egl1* (endoglucanase I), and *egl2* (endoglucanase II), were analyzed by real-time quantitative PCR (qPCR) (Fig. 5). In the Δ76238 strain, the expression levels of four cellulase genes (*cbh1*, *cbh2*, *egl1*, and *egl2*) were significantly increased compared to those in *T. reesei* RUT-C30 using glucose as the carbon source. These results were consistent with the enhanced cellulase production in Δ76238.

In addition, the expression levels of *cbh1*, *cbh2*, *egl1*, and *egl2* in Δ76238 were significantly increased compared with those in *T. reesei* RUT-C30 using lactose as the carbon source (Additional file 1: Fig. S7).

The transcriptional activator *xyr1* is an essential activator controlling the expression of the major cellulase genes [15]. The *xyr1* expression level was measured by qPCR (Fig. 5e). Interestingly, *xyr1* continuously maintained a high expression level from 72 to 120 h in Δ76238 using glucose as the carbon source, indicating that the Δ76238 had a different *xyr1* expression pattern compared with that of *T. reesei* RUT-C30. From 96 to 120 h, the *xyr1* transcription level in Δ76238 was much higher than that in *T. reesei* RUT-C30. The possible reasons may involve significantly different expression patterns of cellulase genes and the main transcription factor *xyr1* in Δ76238.

### Ca^2+^channels are essential for cellulase production in Δ76238 using glucose as the carbon source

Aside from H^+^-ATPase, proton exchangers are also associated with proton and ion homeostasis, such as the Ca^2+^/H^+^ exchanger [38]. A“H^+^-gated pathway” demonstrated that pH variations allow Ca^2+^ transportation via the Ca^2+^/H^+^ exchanger [39]. We speculated that acid accumulation in Δ76238 hindered the growth of the strain. Ca^2+^/H^+^ exchange may also have played an important role in transporting protons out of the Δ76238 cells, along with transporting Ca^2+^ into the cytoplasm, to cause a Ca^2+^ burst in Δ76238 cells. Moreover, an appropriate Ca^2+^ concentration markedly stimulated the Ca^2+^ signal transduction pathway [40], which activated Crz1 (calcineurin-responsive zinc finger transcription factor 1) to induce cellulase expression in *T. reesei* [41, 42].

**Fig. 6:**
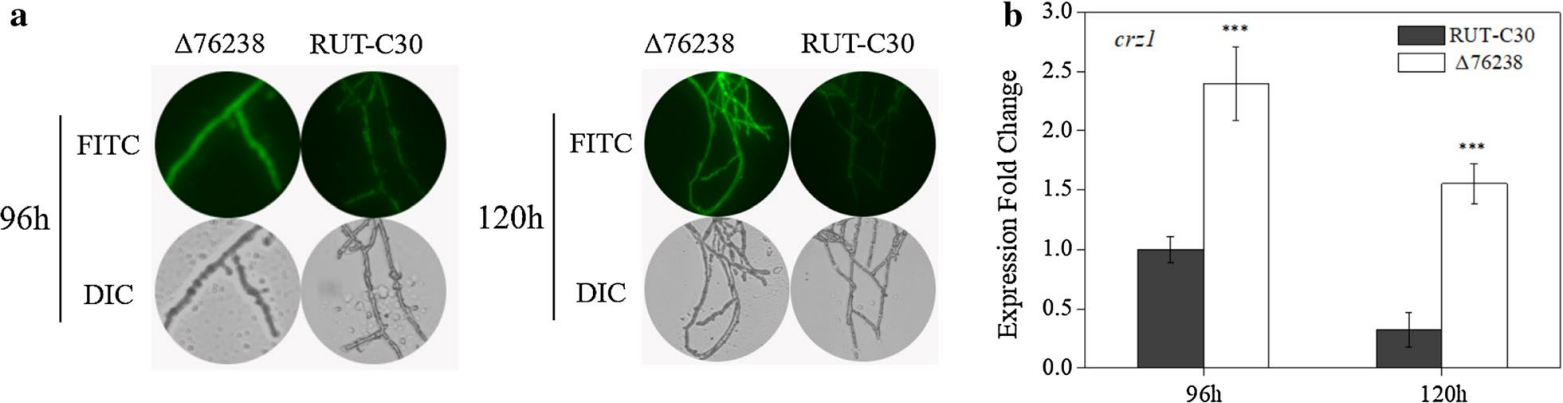
Cytosolic Ca^2+^ levels and the transcription level of the final calcium signaling gene *crz1*. **a** Analysis of cytosolic Ca^2+^ levels via the Ca^2+^ fluorescent probe Fluo-3/AM. *T. reesei* Δ76238 and parental strain RUT-C30 were cultured in MA medium for 96–120 h, using glucose as the sole carbon source. For detection, 50 μM Fluo-3/AM was used, and its intensity was monitored using Automatic Inverted Fluorescence Microscopy. Green fluorescence represents free cytosolic Ca^2+^. **b** Transcriptional level of the final calcium signaling gene *crz1* was evaluated by quantitative real-time PCR (qPCR). *T. reesei* strains were grown using glucose for 96 or 120 h. The data were normalized to the expression of RUT-C30 over 96 h, with the *sar* gene used as an endogenous control in all samples. Values are the mean ± SD of the results from three independent experiments. Asterisks indicate significant differences (***p < 0.001, Student’s *t* test)

We, therefore, investigated the cytosolic Ca^2+^ level in Δ76238 with glucose as the sole carbon source. We used the Fluo-3/AM fluorescent dye [43], in which the green fluorescence intensity represents the relative amounts of free cytosolic Ca^2+^ [41]. As shown in Fig. 6a, a stronger green fluorescence intensity was observed in Δ76238 compared with that in the parental strain *T. reesei* RUT-C30 at 96 h and 120 h, demonstrating that the level of cytosolic Ca^2+^ in Δ76238 was significantly increased compared to that in *T. reesei* RUT-C30.

Cytosolic Ca^2+^ is one of the secondary messengers that induces calcium signaling to trigger the downstream pathway [44]. Our previous work involving Mn^2+^ elicitation and DMF induction showed that an increased cytosolic Ca^2+^ concentration can trigger calcium signal transduction pathways and induce cellulase expression in *T. reesei* [41, 42]. Cellulase production stimulated by low intracellular pH may be similar to that of Mn^2+^ and DMF stimulation [18]. To test our hypothesis, the expression levels of the final calcium signaling gene *crz1* (encoding Crz1) were quantitatively determined by qPCR. With glucose as the sole carbon source, the transcription level of *crz1* in Δ76238 was markedly increased in Δ76238 compared with that in the parental strain *T. reesei* RUT-C30 (Fig. 6b). This demonstrated that the increased level of cytosolic Ca^2+^ is one reason for increased cellulase production in Δ76238.

**Fig. 7:**
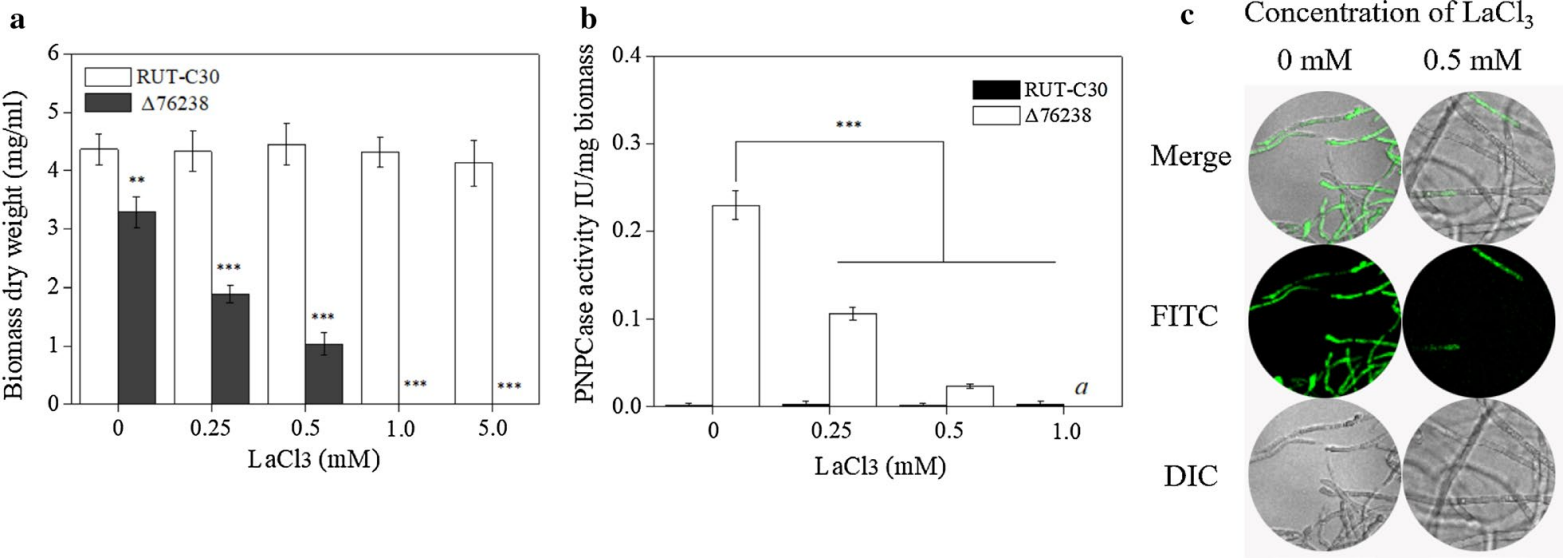
Effects of Ca^2+^ channel inhibitor LaCl_3_ on growth, cytosolic Ca^2+^ concentration, and cellulase production. **a** Biomass dry weight of *T. reesei* RUT-C30 and Δ76238 was measured in 100 mL MA medium treated with different concentrations of LaCl_3_ for 120 h, using glucose as the sole carbon source. **b** PNPCase activity of Δ76238 was examined after culture in MA medium for 120 h containing 0, 0.25, 0.5, or 1 mM LaCl_3_. **c** Fluorescence analysis of LaCl_3_ influence on cytosolic Ca^2+^ burst in Δ76238. Δ76238 was cultured in MA medium for 120 h and treated with 0 or 0.5 mM LaCl_3_, using glucose as the sole carbon source. For detection, 50 μM Fluo-3/AM was used, and its intensity was monitored using the Nikon A1R confocal microscope. Green fluorescence represents the free cytosolic Ca^2+^. “*a*” in the figure indicates that conidia of Δ76238 cannot germinate with 1 mM LaCl_3_. Values are the mean ± SD of the results from three independent experiments. Asterisks indicate significant differences (**p < 0.01, ***p < 0.001, Student’s *t* test)

To address whether the cytosolic Ca^2+^ burst in Δ76238 mediates enhanced cellulase production when glucose is the sole carbon source, we used LaCl_3_, a plasma membrane Ca^2+^ channel blocker, to prevent influx of external Ca^2+^ [45]. We found that conidia of Δ76238 cannot germinate at 1 mM LaCl_3_ and above (Fig. 7a), whereas the parental strain RUT-C30 can grow well at 5 mM LaCl_3_. As shown in Fig. 7b, when glucose was the sole carbon source, cellulase production per g biomass of Δ76238 was obviously reduced, as LaCl_3_ concentration increased. Only a small amount of green fluorescence was observed in Δ76238 treated with 0.5 mM LaCl_3_ when glucose was the sole carbon source (Fig. 7c). These results confirmed that calcium signaling plays a dominant role in cellulase and biomass production in Δ76238.

### Optimizing the medium promotes the growth of Δ76238 for cellulase production in a jar fermenter

Δ76238 showed significantly increased cellulase production under low-cost carbon source glucose conditions. However, the low growth rate due to intracellular acid accumulation using glucose as the carbon source, hindered its application. Δ76238 growth could be restored by adding appropriate amounts of aqueous ammonia (Additional file 1: Fig. S4). Proton accumulation suppressed Δ76238 growth in the early phase. Therefore, we hope to promote its growth rate by optimizing the medium in the first conidia germination stage. A simple optimization strategy is to use a nitrogen-rich medium as the seed medium, followed by transferring the culture to the fermentation medium using glucose as the sole carbon source, to produce cellulase in a jar fermenter.

**Fig. 8:**
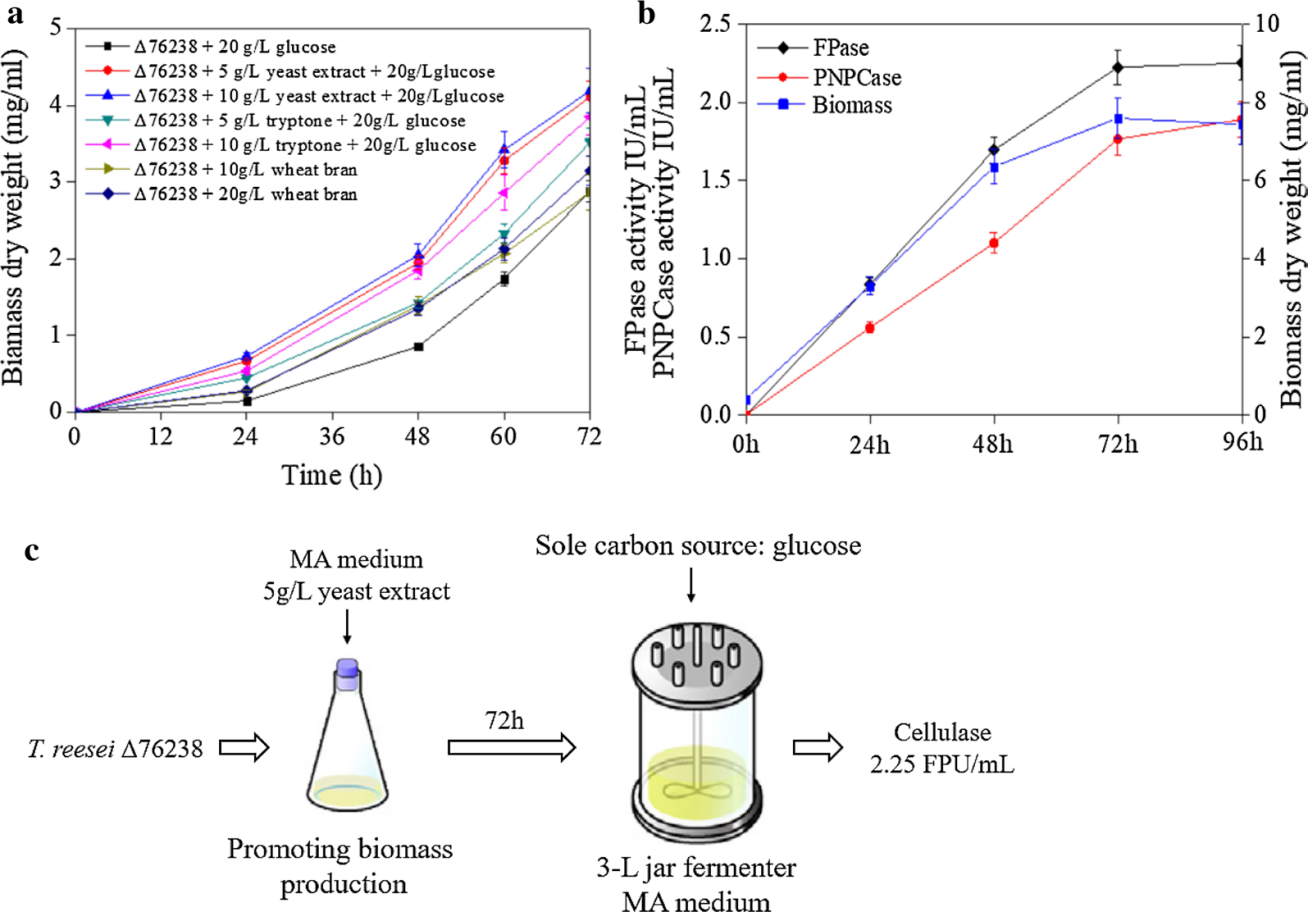
Growth and cellulase production of Δ76238 by optimizing the medium. **a** Biomass dry weight of *T. reesei* strains was measured after being grown in different optimizing seed media, with yeast extract, tryptone, and wheat bran as candidate extra nitrogen sources at different concentrations (0.5% to 2%). **b** FPase activity, PNPCase activity, and biomass dry weight of Δ76238 were measured. Δ76238 was cultivated in 100 mL MA medium containing 5 g/L yeast extract and 20 g/L glucose in the early phase (3 days), and was then transferred to 1 L MA medium containing 20 g/L glucose with 10% inoculation. **c** Flow chart of the process of Δ76238 for cellulase production in a jar fermenter. Values are the mean ± SD of the results from three independent experiments

We initially attempted to choose yeast extract, tryptone, and wheat bran as candidates for extra nitrogen sources, at different concentrations (5 g/L to 20 g/L) added in MA medium. Figure 8a shows that addition of 5 g/L to 10 g/L yeast extract was the best choice for promoting Δ76238 biomass production, and increased the biomass by 45% more than when there was no extra nitrogen addition after 72-h cultivation.

As shown in Fig. 8c of the flow chart, when cultivated in 100 mL MA medium containing 5 g/L extra yeast extract for 72 h, the Δ76238 biomass was collected by filtering and was transferred to a 3 L jar fermenter. This had a final working volume of 1 L MA medium, containing 2% (w/v) glucose as the sole carbon source for cellulase production. The FPase and PNPCase activities of Δ76238 had reached high levels on the 2nd day (Fig. 8b). Therefore, Δ76238 may be feasible for amplifying fermentation (Fig. 8c).

## Discussion

In this study, we investigated the influence of intracellular pH on cellulase production by *T. reesei*. Plasma membrane H^+^-ATPase from fungi is a proton pump that plays a key role in intracellular pH regulation [23, 28, 35]. In this study, two H^+^-ATPase genes, *tre76238* and *tre78757*, were researched in *T. reesei*. Our results initially demonstrated that the H^+^-ATPase gene *tre76238* is involved in cellulase induction in *T. reesei*, and that cellulase can be produced in the repressive carbon source glucose culture conditions. This extends our knowledge of cellulase regulation.

The hypersecreting mutant *T. reesei* RUT-C30 was selected as the parental strain for H^+^-ATPase gene knockout due to its defective carbon catabolite repression phenotype (mutated cre1). Carbon catabolite repression (CCR) in *T. reesei* is the means by which cells manage the priority use of glucose over more complex molecules such as cellulose. Although *T. reesei* RUT-C30 carries a truncated form of CREI and has a general loss of CREI-mediated carbon catabolite repression, *T. reesei* RUT-C30 produces very low levels of cellulases with glucose as the sole carbon source [46]. The mutant Δ76238 was constructed by deletion of *tre76238* in *T. reesei* RUT-C30. Δ76238 showed retardation in strain growth and significant cellulase production, with glucose as the sole carbon source, compared with the parental strain *T. reesei* RUT-C30. Deletion of *tre76238* in mutant Δ76238 was re-complemented by transforming the wild-type *tre76238* into its genome. The resulting complementation strain R76238 was obtained and demonstrated the restoration of growth and enzyme activities to levels similar to those of the starting strain *T. reesei* RUT-C30 (Additional file 1: Fig. S8). This demonstrated that gene knockout of *tre76238* contributed to growth retardation and improved cellulase production in Δ76238 using glucose as the carbon source.

We also deleted *tre76238* in the wild-type strain *T. reesei* QM6a and another mutant, *T. reesei* QM9414, which is closest to the wild-type strain. However, no cellulase production phenotype was detected under conditions of glucose as the sole carbon source except growth delay (Additional file 1: Fig. S9). The intact *cre1* in both *T. reesei* QM6a and QM9414 might be the main reason for the lack of a cellulase production phenotype using glucose as the carbon source, which requires further study.

The growth rate of deletion strains showed that *tre76238* plays a major role and *tre78757* plays a minor role as plasma membrane H^+^-ATPases in *T. reesei*. The *tre78757* gene, a functionally interchangeable isogene of *tre76238*, is dispensable for growth and is an additional function for *tre76238*. When *tre76238* was deleted from *T. reesei*, *tre78757* functioned as a plasma membrane H^+^-ATPase in *T. reesei*. Similarly, of the two H^+^-ATPase isogenes *pma1* and *pma2* in *S. cerevisiae*, *pma1* is essential for growth, whereas *pma2* is dispensable [28]. Therefore, according to the gene functions, the H^+^-ATPase genes *tre76238* and *tre78757* were separately named for *pma1* and *pma2* in *T. reesei*.

We explored the reasons for significant cellulase production in Δ76238 using glucose as the carbon source. First, we analyzed the transcriptional levels of the main factor *xyr1*. *xyr1* transcription in *T. reesei* RUT-C30 can occur under conditions of glucose as a cellulase-repressive carbon source due to the lack of *cre1* [47]. Stricker et al. [47] reported that the transcription level of *xyr1* in *T. reesei* RUT-C30 in these glucose conditions gradually decreased with time, whereas the transcription level of *xyr1* under conditions of lactose as a cellulase-inducing carbon source was continuous. Meanwhile, *xyr1* was continuously transcribed in Δ76238 under conditions of glucose as a repressive carbon source (Fig. 5), which is quite different from that in the parental strain *T. reesei* RUT-C30.

Second, we analyzed the concentration of cytosolic Ca^2+^. Zhai et al. [48] reported that Ca^2+^ is transported in *Arabidopsis* cells, mainly through a Ca^2+^/H^+^ exchange system driven by the proton-motive force from plasma membrane H^+^-ATPase. As reported by Inesi et al. [38], H^+^ concentration reduction by conditions of high pH prevented Ca^2+^/H^+^ exchange. Deleting the H^+^-ATPase gene *tre76238* in *T. reesei* impaired the transportation of protons out of cells, and significantly increased the concentration of intracellular protons (Fig. 3). Significantly increased intracellular Ca^2+^ levels were found in Δ76238 (Fig. 6). Chen et al. [41] reported that a cytosolic Ca^2+^ burst can markedly stimulate *T. reesei* cellulase production. The increased Ca^2+^ level was demonstrated to be necessary for increasing cellulase production in Δ76238. Δ76238 showed no cytosolic Ca^2+^ burst and could not produce cellulase using glucose as the carbon source after the Ca^2+^ channel blocker LaCl_3_ was added (Fig. 7). Plasma membrane Ca^2+^ channels can also affect the intracellular pH [38, 39]. High LaCl_3_ concentrations (> 1 mM) completely hindered the growth of Δ76238 (Fig. 7a). Therefore, calcium signaling was demonstrated to play a dominant role in Δ76238 cellulase and biomass production.

**Fig. 9:**
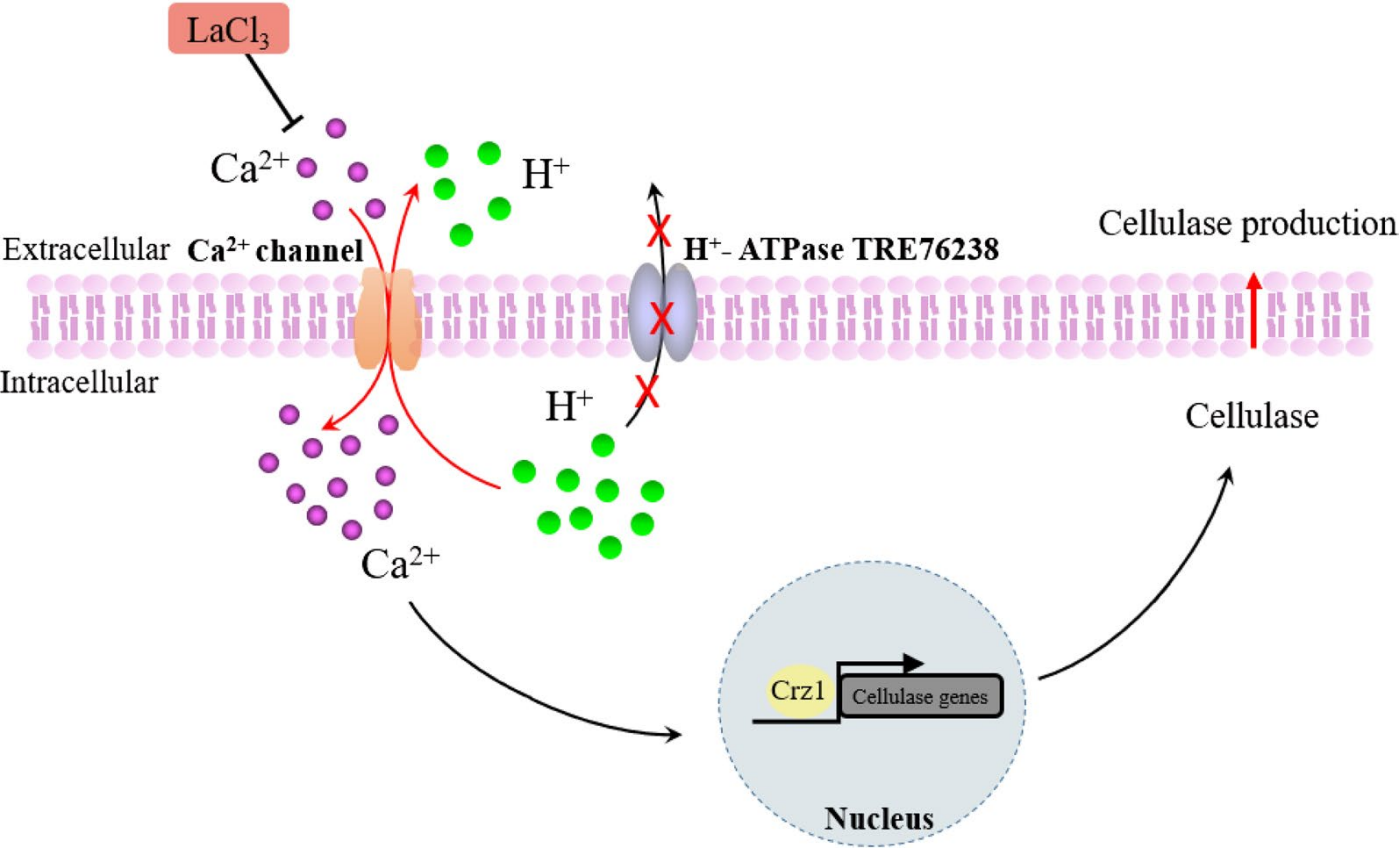
Mechanistic model for decreasing intercellular pH by deleting the H^+^-ATPase gene *tre76238*, thus enhancing cellulase production in *T. reesei.* Deleting *tre76238* impairs the ability of protons to be transported out of cells. Intracellular acid accumulation promotes a cytosolic Ca^2+^ burst that activates calcium signaling. The effects of adding LaCl_3_ suggest that Ca^2+^ channels are essential for cellulase production in Δ76238, using glucose as a repressive carbon source. Solid arrows indicate the data supported by our own experiments

These results imply a mechanistic model of cellulase production in Δ76238 using glucose as the carbon source (Fig. 9). In Δ76238, it was difficult for intracellular protons to be exported from cells due to the defective function of H^+^-ATPase gene *tre76238*. Instead, Ca^2+^ channels, such as Ca^2+^/H^+^ exchangers [39], play an important role in transporting protons out of Δ76238 and balancing the intracellular pH. Intracellular acid accumulation in Δ76238 produced a proton gradient, and large pH variations allowed Ca^2+^ transportation by the Ca^2+^/H^+^ exchanger [39]. This led to a cytosolic Ca^2+^ burst that activated the calcium signal transduction pathway, inducing cellulase production in *T. reesei* (Fig. 6). We, therefore, carried out a BLAST search of the *Trichoderma reesei* (https://genome.jgi.doe.gov/pages/blast-query.jsf?db=Trire2) genome sequence for genetic matches to the Ca^2+^/H^+^ exchanger genes from some organisms, including *Saccharomyces cerevisiae* [49] and *Escherichia coli* [50]. Seven putative Ca^2+^/H^+^ exchanger genes (*tre79599*, *tre55595*, *tre79398*, *tre56744*, *tre82544*, *tre62835*, and *tre68169*) were identified in *T. reesei*; however, understanding their functions in Ca^2+^ transportation warrants further study. Moreover, knockout of these Ca^2+^/H^+^ exchanger genes may also change the intracellular pH in *T. reesei*, as occurs in mammalian cells [39].

Intracellular acid accumulation totally changed the intracellular environment, making cellulase production with glucose as a repressive carbon source, a more complicated process. Other reasons for enhancing Δ76238 cellulase production using glucose still need to be further researched, and may be meaningful for improving industrial cellulase production using *T. reesei*. Earlier studies have shown that many *T. reesei* intracellular *β*-glucosidases catalyze transglycosylation, glycosylating glucose to generate sophorose [51], which is the most effective soluble inducer for cellulase production [52]. Proton accumulation in Δ76238 might activate the intracellular transglycosylation reaction to form the tiny cellulase inducer sophorose, thus inducing cellulase production in Δ76238.

Our study demonstrated that Δ76238 growth can be accelerated in the early phase, and cellulase production can be stable after transfer to a scale-up culture in new medium. Therefore, Δ76238 has the potential for industrial cellulase production using glucose as a low-cost carbon source. Depending on the results of this study, we predicted whether establishment of a controlled switch for *tre76238* transcription downregulation in *T. reesei* RUT-C30 would allow normal growth during the early phase of fermentation with the switch turned on. The strain can then produce a large amount of cellulase during the late phase of fermentation with the switch turned off. This kind of smart *T. reesei* strain may have potential for industrial application.

## Conclusions

In summary, this study reports a H^+^-ATPase gene deletion strain Δ76238 with greatly enhanced cellulase production in addition to intracellular acid accumulation and growth retardation using cellulase-repressive glucose as the sole carbon source (Fig. 9). Our results indicate that intracellular acid accumulation in Δ76238 stimulated a significant increase in the levels of cytosolic Ca^2+^. It also triggered Ca^2+^-CRZ1 signaling to induce cellulase gene transcription using glucose as the carbon source. Ca^2+^channels were demonstrated to be necessary for cellulase production using glucose as the carbon source in Δ76238. Δ76238 growth delay could be reversed by optimizing the medium’s nitrogen sources to produce ammonia. This allowed neutralization of intracellular acid in the early phase, which may be used for scale-up cellulase production using glucose as the carbon source. This study provides a new perspective for greatly altering the cellulase expression pattern in *T. reesei* Δ76238, and demonstrates a new mechanism for cellulase regulation in conditions of low intracellular pH.

## Methods

### Strains and culture conditions

The *T. reesei* hosts used in this research include QM9414 (ATCC 26921) and RUT-C30 (ATCC 56765). Spore suspensions were prepared by cultivating the fungus on potato-dextrose plates, after which the spores were harvested, suspended in a buffer containing 0.8% NaCl, 0.025% Tween 20 and 20% glycerol, and stored at − 80 °C.

To analyze enzyme production, conidia (final concentration 10^6^/mL) of *T. reesei* strains were grown at 28 °C, in 100 mL of MA medium containing 2% (w/v) Avicel (PH-101, Sigma-Aldrich), 2% (w/v) lactose, or 2% (w/v) glucose as the sole carbon source [53]. Mycelia were collected at different time intervals and kept frozen at − 80 °C for RNA extraction. The supernatant was used for enzyme assays.

### Biomass concentration assay

For biomass analysis, the mycelium from lactose and glucose was harvested using pre-weighed glass microfiber filters (Cat. No. 1822-055, Whatman, Kent, UK) at appropriate time intervals, washed with water, dried at 65 °C for one day, and analyzed. The biomass dry weight from Avicel was measured as described by Zhang et al. [53]. Each experiment was performed in three biological replicates.

### Vector construction and transformation

*Trichoderma reesei* RUT-C30 lacking *tku70* [54] was used as the recipient for all targeted gene knockouts. Deletion cassettes for selected genes were constructed by ligating 0.9 to 1 kb 5′- and 3′-flanks of each gene to the hygromycin-resistant plasmid LML2.1 [54]. As shown in Additional file 1: Fig. S10, the upstream fragment was ligated to the *Pac*I- and *Xba*I-linearized LML2.1 using the ClonExpress™ II One Step Cloning Kit (Vazyme, Nanjing, China). Subsequently, the downstream fragment was inserted into the SwaI site to form the deletion cassettes. The re-complementation cassettes of the genes were constructed by ligating whole gene sequences (including the 1.5 kb promoter, gene coding sequence, and 1 kb terminator) to LML2.1. Re-complementation cassettes were transformed into the corresponding gene knockout mutants as described previously [41].

Deletion cassettes were transformed into *T. reesei* by *Agrobacterium*-mediated transformation [55]. Re-complementation cassettes were transformed into the corresponding gene knockout mutants by *Agrobacterium*-mediated transformation [41]. The hygromycin-resistant cassette can be self-excised by xylose-induced Cre recombinase [54], if necessary. The putative gene disruption mutants generated by double crossover were verified by diagnostic PCR using the primers XX-CF and XX-CR (XX represents the gene name). The fragments generated from the genome of transformants by PCR, using the primers XX-CF and XX-CR, were sequenced to verify the correct transformants. The primers used in this study are shown in Additional file 1: Table S1.

### Bio-informatics analysis and phylogenetic analysis

Protein sequences were obtained from the Joint Genome Institute of Department of Energy (USDOE-JGI) website (http://genome.jgipsf.org/Trire2/). Homologs of *T. reesei* proteins were identified using BLASTP (http://blast.ncbi.nlm.nih.gov/), with an E value < 10^−5^ applied as a cutoff. Putative transmembrane regions were predicted by the TMHMM program v.2.0 (http://www.cbs.dtu.dk/services/TMHMM-2.0/). Protein modeling was performed using the PHYRE2 server [34]. Alignments of multiple protein sequences were performed using MUSCLE [56]. Phylogenetic analysis of protein was carried out in MEGA6 using the neighbor-joining method with 1000 bootstrap replicates.

### Cellulase production in a jar fermenter

For promoting the growth of *T. reesei* strains using glucose as the sole carbon source, MA medium was optimized by separately adding different nitrogen sources (5 g/L or 10 g/L yeast extract, 5 g/L or 10 g/L tryptone, 10 g/L or 20 g/L wheat bran). After determining the best nitrogen source for growth, *T. reesei* strains were cultured in 100 mL optimized MA medium in the early phase (3 days), adding 5 g/L yeast extract. The strains were washed and transferred into a 3 L jar fermenter (Baoxing BIO-ENGINEERING EQUIPMENT, shanghai, China) with a final working volume of 1 L MA medium, containing 2% (w/v) glucose with 10% inoculation for the late phase of cellulase production. The supernatant was used for enzyme assays.

### RNA isolation and real-time quantitative PCR (RT-qPCR)

The levels of gene-specific mRNA were assessed using RT-qPCR as per our previous study [54]. In brief, total RNA was carefully extracted from frozen mycelia using a FastRNA Pro Red Kit (MPbio, Irvine, CA, USA) as per the manufacturer’s instructions. Total RNA (500 ng) was reverse-transcribed and cDNA was synthesized using the PrimeScript RT Reagent Kit with gDNA eraser (TaKaRa, Japan), according to the manufacturer’s instructions. qPCR was performed using an ABI StepOne thermocycler (Applied Biosystems, Foster City, CA, USA) and the TransStart TipTop Green qPCR SuperMix (TransGen, Shanghai, China) with 200 nM of forward and reverse primers (Additional file 1: Table S1). Thermal cycling was conducted under the following conditions: an initial denaturation step at 95 °C, followed by 40 amplification cycles of 5 s at 94 °C and 60 s at 64 °C. For transcription analysis, an SYBR green assay with reference to the *sar1* gene was performed [57]. Melt curves were obtained after each RT-qPCR run to confirm the specificity of amplification and the absence of primer dimers.

### Enzyme activity assays

Filter paper hydrolase (FPase) activity, representing total extracellular cellulase activity, was determined using the 3, 5-dinitrosalicylic acid method [58].

Cellobiohydrolase activity (*p*NPCase) was determined using 5 mM *p*-nitrophenol-d-cellobioside (Sigma-Aldrich) as a substrate in 50 mM sodium acetate buffer at pH 5.0 [53]. The release of *p*-nitrophenol was assessed by measuring absorbance at 405 nm. One unit of enzymatic activity was defined as 1 μmol of *p*-nitrophenol released from the substrate per minute.

### Fluorescence detection

Intracellular pH was detected using the fluorescent pH probe BCECF-AM (pKa: 6.98, working range is 6.0–8.0) [36]. Living cell green fluorescence was measured using an S Plan Fluor ELWD 40×, 0.45 numerical aperture (NA) objective and a digital sight camera on an Eclipse Ti inverted Nikon, using a FITC filter with excitation filters from 420 nm to 490 nm, 10 nm intervals, and an emission filter of 535 nm.

Mitochondrial activity was detected using MitoTracker^®^ Red CMXRos, using a Texas Red filter with excitation filters from 500 nm to 620 nm, 10 nm intervals, and an emission filter of 670 nm. The fluorescence was resolved from background fluorescence using the CareStream Multispectral program.

Fluo-3/AM (Sigma) was used to assess the level of cytoplasmic Ca^2+^ in *T. reesei* according to the manufacturer’s protocol. Fluo-3/AM (50 μM final concentration) was loaded into cells by incubation at 37 °C for 30 min, and the cells were then washed thrice with phosphate-buffered saline. Images of Ca^2+^ green fluorescence were obtained using an S Plan Fluor ELWD 40x, 0.45 numerical aperture (NA) objective and a digital sight camera on an Eclipse Ti inverted Nikon or Nikon A1R confocal microscope, comprising an FITC filter (420–490 nm band-pass excitation filter, and emission filter of 535 nm). The intensity of green fluorescence was quantified using NIS-Elements F package software. To eliminate the contribution of background fluorescence, cells without Fluo-3 AM labeling were also imaged under identical conditions.

### Statistical analysis

All experiments were carried out at least three times with identical or similar results. The error values indicate the standard deviation (SD) from the mean of triplicates. Statistical significance was calculated using Student’s *t* test analysis with a significance of *p* < 0.05.

## Additional file

**Additional file 1: Table S1.** Primers used in this study. **Fig. S1.** Structural views of TRE76238 and TRE78757. **Fig. S2.** Mycelial morphology of Δ76238 and *T. reesei* RUT-C30. **Fig. S3.** The external pH, intracellular pH, and mitochondrial activity of *T. reesei* strains. **Fig. S4.** The growth of Δ76238 when adding aqueous ammonia. **Fig. S5.** Δ76238 cellulase production using Avicel as the carbon source. **Fig. S6.** Cellulase production of *T. reesei* Δ78757. **Fig. S7.** Transcription levels of major cellulase-related genes in *T. reesei* Δ76238 using lactose as the carbon source. **Fig. S8.** The growth and PNPC activity of the complementation strain R76238. **Fig. S9.** The growth of Q76238, Q78757, and QM9414 strains. **Fig. S10.** Construction of deletion mutants.

## Data Availability

All data generated or analyzed during this study are included in this published article and its additional files.

## Abbreviations

crz1: calcineurin-responsive zinc finger transcription factor 1
FPase: filter paper hydrolase activity, representing total extracellular cellulase activity
PNPCase: exo-β-glucanase activity
qPCR: quantitative PCR
