# Supplementary material for: Enhanced cellulase production by decreasing intercellular pH through H+-ATPase gene deletion in Trichoderma reesei RUT-C30
Source: Biotechnol Biofuels. 2019 Aug 13;12:195. doi: 10.1186/s13068-019-1536-2 (PMC6691542; doi:10.1186/s13068-019-1536-2)
Supplement: Supplementary file 1 — Additional file 1: Table S1. Primers used in this study. Fig. S1. Structural views of TRE76238 and TRE78757. Fig. S2. Mycelial morphology of Δ76238 and T. reesei RUT-C30. Fig. S3. The external pH, intracellular pH, and mitochondrial activity of T. reesei strains. Fig. S4. The growth of Δ76238 when adding aqueous ammonia. Fig. S5. Δ76238 cellulase production using Avicel as the carbon source. Fig. S6. Cellulase production of T. reesei Δ78757. Fig. S7. Transcription levels of major cellulase-related genes in T. reesei Δ76238 using lactose as the carbon source. Fig. S8. The growth and PNPC activity of the complementation strain R76238. Fig. S9. The growth of Q76238, Q78757, and QM9414 strains. Fig. S10. Construction of deletion mutants. [file 13068_2019_1536_MOESM1_ESM.docx]

**Figure captions**

**Fig. S1.** **Structural views of TRE76238 and TRE78757.** A and B are structural views of the protein TRE76238 and TRE78757. The sequences of proteins TRE76238 and TRE78757 were submitted to the PHYRE2 server for modeling. The highest confidence model was shown and was based on the *Neurospora crassa* plasma membrane H^+^-ATPase (PDB c1mhsA). The model was visualized using JSmol, and the approximate locations of the cytosolic actuator (A), nucleotide-binding (N), phosphorylation (P) and c-terminal (CT) domains are indicated based on comparison to the yeast Pma1 structure.

**Fig. S2.** **Mycelial morphology of Δ76238 and *T. reesei* RUT-C30.** *T. reesei* strains were cultivated in 100 mL MA medium using glucose, lactose, and Avicel as the sole carbon source, and part of hyphae in the fermentation broth was transferred into culture dishes for taking photos.

**Fig. S3.** **The external pH, intracellular pH, and mitochondrial activity of *T. reesei* strains.** A and B, External pH was measured in Δ78757 and parental strain *T. reesei* RUT-C30 containing glucose (A), lactose (B) as the carbon source. External pH was measured by pH electrode. C and D, Intracellular pH of Δ78757 and *T. reesei* RUT-C30 was characterized in 72 h and 96 h using glucose (C) and lactose (D) as the sole carbon source. Mycelia of *T. reesei* strains were labeled with 5 μM pH fluorescent probe BCECF-AM for 30 min. E, Mitochondrial activity of Δ78757 and *T. reesei* RUT-C30 was measured using glucose or lactose as the carbon source at 96 h. Mycelia of *T. reesei* strains were stained with 100 nM MitoTracker® Red CMXRos for 30 min.Values are the mean ± SD of the results from three independent experiments.

**Fig. S4. The growth of Δ76238 when adding aqueous ammonia.** 100 ul of aqueous ammonia (25%, w/v) was added into the fermentation broth of Δ76238 in 24 h cultured in 100 mL MA medium using glucose as the sole carbon source. Biomass dry weight of Δ76238 with/without aqueous ammonia was measured in 48h. Values are the mean ± SD of the results from three independent experiments. Asterisks indicate significant differences (***p < 0.001, Student’s *t-*test).

**Fig. S5. Δ76238 cellulase production using Avicel as the carbon source.** The FPase (A) and PNPCase (B) activities of Δ76238 were measured compared with that of *T. reesei* RUT-C30 using Avicel as the sole carbon source. Values are the mean ± SD of the results from three independent experiments.

**Fig. S6.** **Cellulase production of *T. reesei* Δ78757.** The FPase activities of Δ78757 were separately measured compared with that of RUT-C30 using glucose (A), lactose (B), or Avicel (C) as the sole carbon source. The PNPCase activities of Δ78757 were separately measured compared with that of RUT-C30 using glucose (D), lactose (E), or Avicel (F) as the sole carbon source. Values are the mean ± SD of the results from three independent experiments.

**Fig. S7. Transcription levels of major cellulase-related genes in *T. reesei* Δ76238 using lactose as the carbon source.** The transcriptional levels of major cellulase genes *cbh1* (A), *cbh2* (B), *egl1* (C), *egl2* (D), and *xyr1*(E) were evaluated by quantitative real-time PCR (qPCR). *T. reesei* strains were grown on lactose for 72, 96, or 120 h. The data are normalized to the expression of RUT-C30 at 72 h for each tested gene, with *sar* gene used as an endogenous control in all samples. Values are the mean ± SD of the results from three independent experiments. Asterisks indicate significant differences (**p < 0.01, ***p < 0.001, Student’s *t-*test).

**Fig. S8.** **The growth and PNPC activity of the complementation strain R76238.** Biomass dry weight and PNPC activity of *T. reesei* strains RUT-C30 and R76238 were measured in 100 mL MA medium using glucose as the sole carbon source. Values are the mean ± SD of the results from three independent experiments.

**Fig. S9. The growth of Q76238, Q78757, and QM9414 strains.** Biomass dry weight of *T. reesei* strains was measured in 100 mL MA medium using glucose (A), lactose (B), and Avicel (C) as the sole carbon source. Values are the mean ± SD of the results from three independent experiments. The significance was analyzed between the biomass dry weight of *T. reesei* R76238 and QM9414. Asterisks indicate significant differences (**p < 0.01, ***p < 0.001, Student’s *t-*test).

**Fig. S10.** **Construction of deletion mutants.** Schematic representation of the selected genes locus from RUT-C30 and deleting mutants. Deletion cassettes for selected genes were constructed by ligating 0.9 to 1 kb 5′- and 3′-flanks of each gene to the hygromycin resistant plasmid LML2.1.

**Table S1 Primers used in this study.**

| Primer | oligos Sequences (5’ to 3’) |
| --- | --- |
| **Quantitative RT-PCR analysis** | |
| cbh1-q1 | CTCCATCTCCGAGGCTCTTACC |
| cbh1-q2 | GCAAGTGCCGCCATATCTGTTAT |
| cbh2-q1 | GCATATTACGCCTCTGAAGTTAGCA |
| cbh2-q2 | GCATAGTTACCGCCATTCTTGTTG |
| egl1-q1 | GCAGCCTCACCATGAACCAGTA |
| egl1-q2 | CACCGTCAGAGTCCAGGAGATAC |
| egl2-q1 | TGAACAAGTCCGTGGCTCCAT |
| egl2-q2 | ACAATTCGTAGGTCCGCTCCAA |
| hac1-q1 | GCTTCGCCCAACGCCTCAAC |
| hac1-q2 | TCCGCAGCGGCATAGTTGCT |
| xyr1-q1 | CCGGGCTCGCTGAACATGGA |
| xyr1-q2 | CTGGCGCCCTTGTACTCGCT |
| sar-q1 | TGGATCGTCAACTGGTTCTACGA |
| sar-q2 | GCATGTGTAGCAACGTGGTCTTT |
| C20043-q1 | GTTCAAGCGCAAGCACAA |
| C20043-q2 | GCACGAGTGATGGAGGTGT |
| Tbip-q1 | GACGAGGCTGTTGCTTTC |
| Tbip-q2 | TCTGGCTCTTGCGAGTGGG |
| crz-q1 | CCAGCAGATGCCGGACACCA |
| crz-q2 | GTGCATATCGCCGCCCATGC |
| **Construction of *tre76238* and *tre78757* gene deletion vectors** | |
| 76238-D1 | *GATTACGAATTCTTAATTAA*TTTCTTCAGGCCGGTGCCA |
| 76238-D2 | *TGCTATACGAATAATTTCTAG*GGCAACTGACAATGGGAAAGCA |
| 76238-D3 | *ACTAGTGAGCTCATTT*TGACGAGCCATTTGCCTACGA |
| 76238-D4 | *AGTGCCAAGCTTATTT*CCGAGTTGTCTCCAGCAGGTT |
| 78757-D1 | *GATTACGAATTCTTAATTAA*TGCTCTGCCGAGTGTATCTCTG |
| 78757-D2 | *TGCTATACGAATAATTTCTAG*TAGGGTTTGGCGTTGGCTGAT |
| 78757-D3 | *ACTAGTGAGCTCATTT*GCAGCGGATGAGAGGCAACT |
| 78757-D4 | *AGTGCCAAGCTTATTT*CAACAGCATCACGGGTCAAGAC |
| **Verification of the *tre76238* and *tre78757* gene deletion mutants** | |
| 76238-CF | GCTCGGCTCTGTGTGTTATTCG |
| 76238-CR | GGAAGCCGCCATCTCTAGTCTC |
| 76238-orf1 | GCTGTACCGTTGTTCGTGATGG |
| 76238-orf2 | GGCGATCTTGAGGTGGTGGAA |
| 78757-CF | GCTGAGAAGGCGAAAGGAAGAG |
| 78757-CR | AACCGCTCTCCTTGCCAACA |
| 78757-orf1 | GCTCAACGGCATTGGTACGATT |
| 787578-orf2 | AACGGCGGTAACCTTCTTGGA |
| D70-4 | TCGGACTTGCGGAGGATGTTGTAT |
| HG3.6 | TGCCTAGTGAATGCTCCGTAACA |
| **Construction of *tre76238* gene re-complementation vector** | |
| 76238rc-1 | *ACTAGTGAGCTCATTT*GCTCGGCTCTGTGTGTTATTCG |
| 76238rc-2 | TCTCCTTGGCGATGGCGATG |
| 76238rc-3 | CATCGCCATCGCCAAGGAGA |
| 76238rc-4 | *AGTGCCAAGCTTATTT*TGCTTCGTGTCTGGAGAAGAGG |


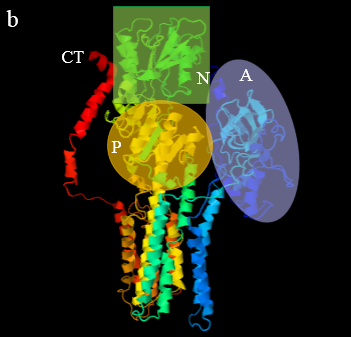

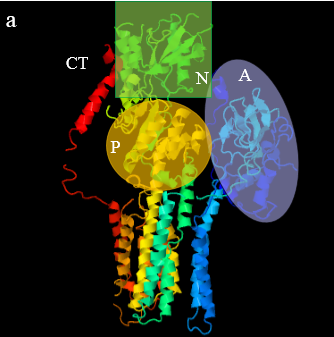
A B

**Fig. S1**


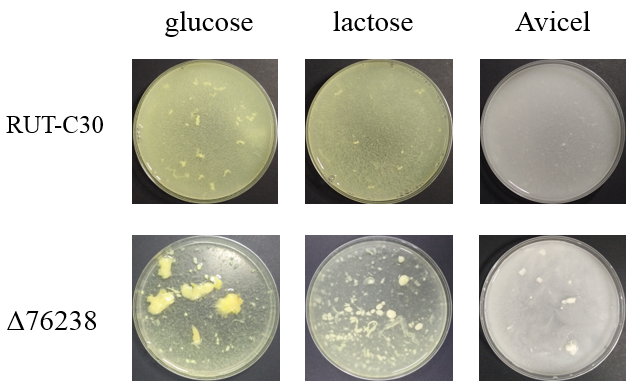


**Fig. S2**


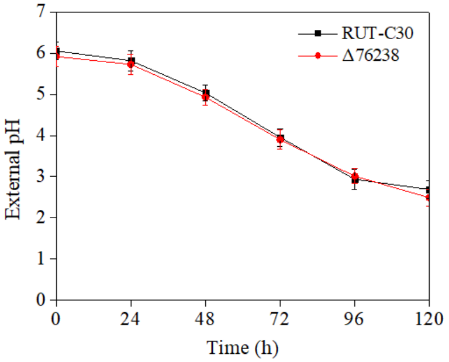

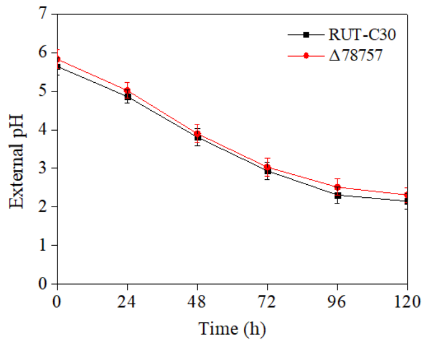
**A B**


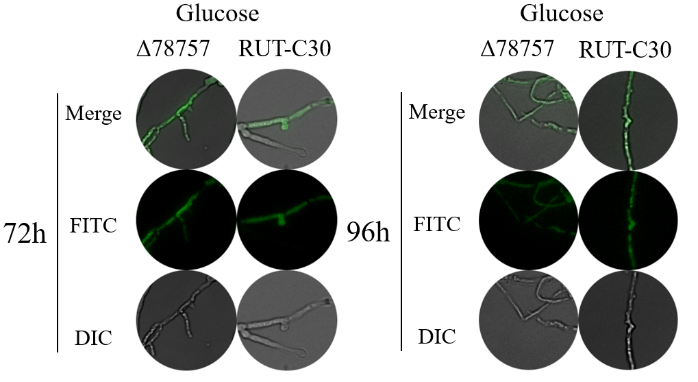
**C**


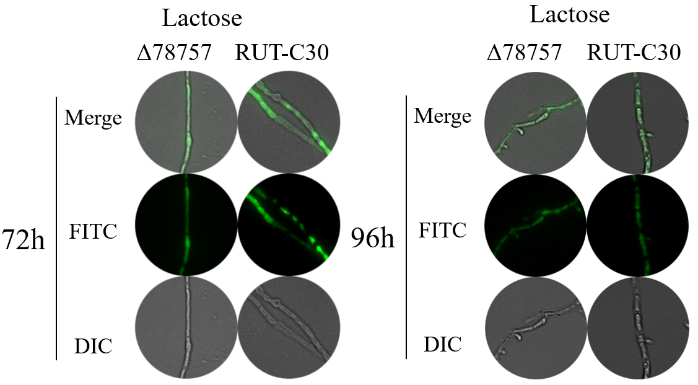
**D**


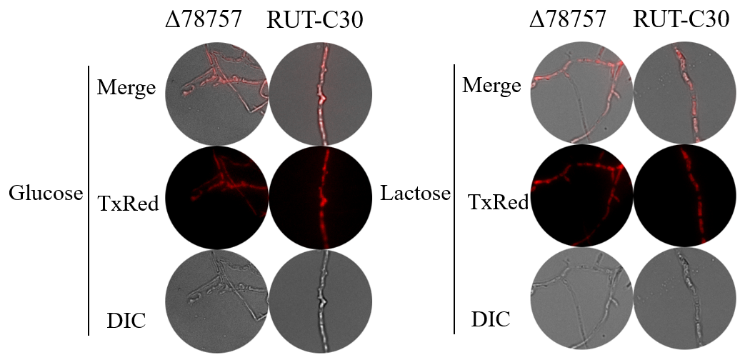
**E**

**Fig. S3.**


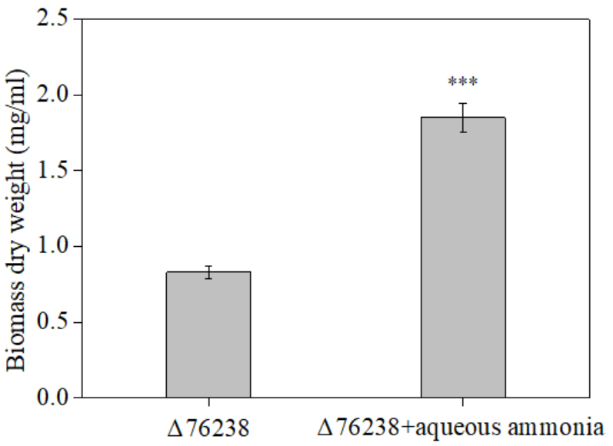


**Fig. S4**


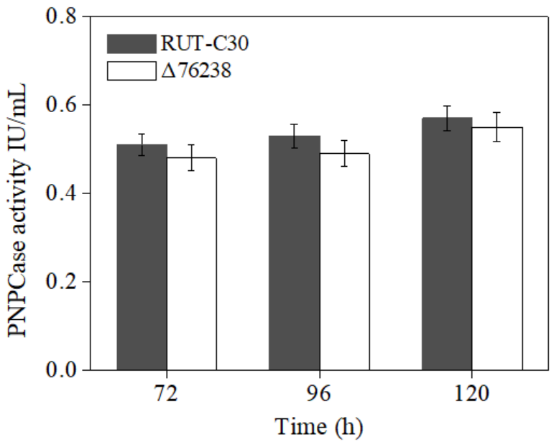

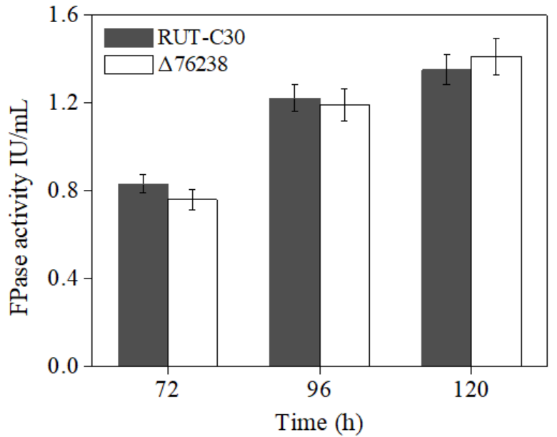
A B

**Fig. S5**


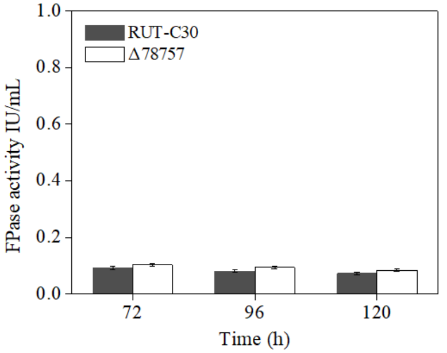

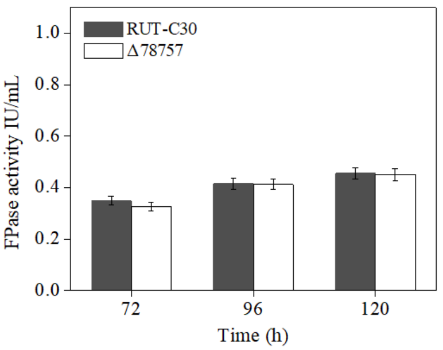

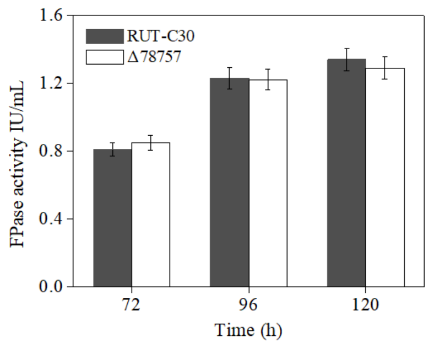
A B C


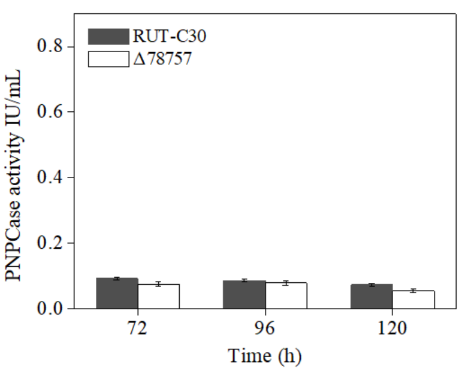

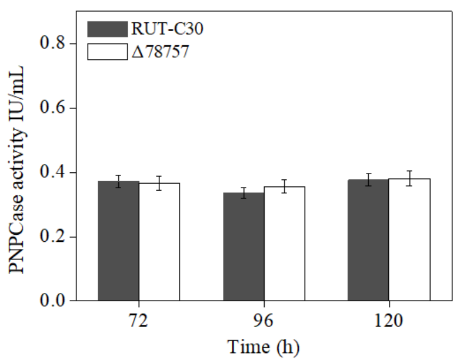

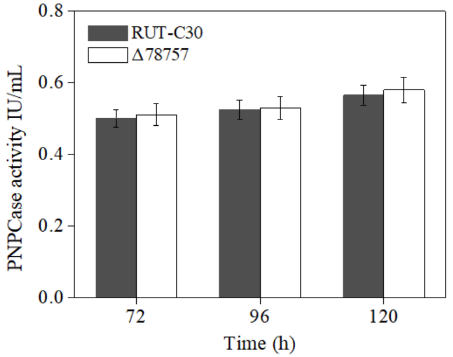
C D E

**Fig. S6**


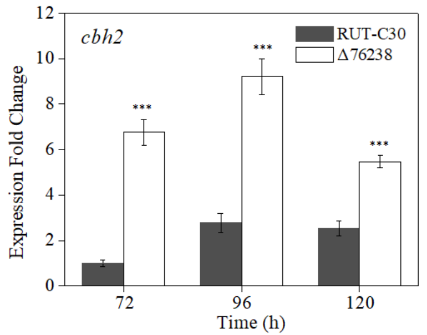

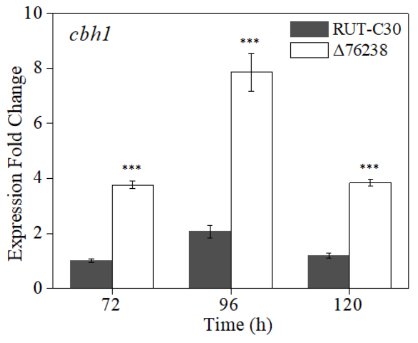

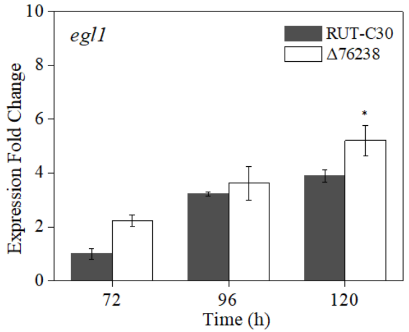
A B C


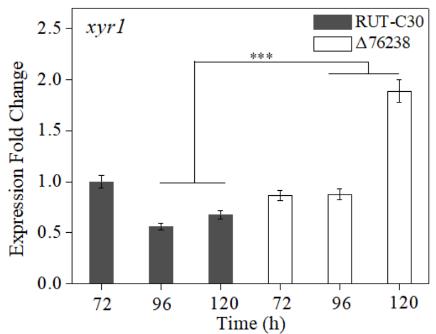

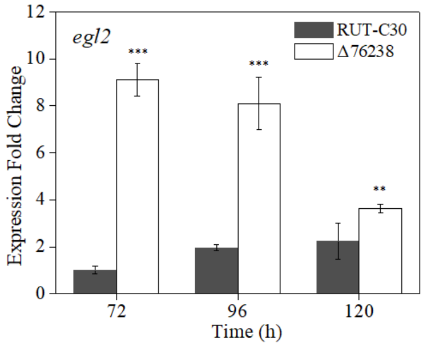
D E

**Fig. S7**


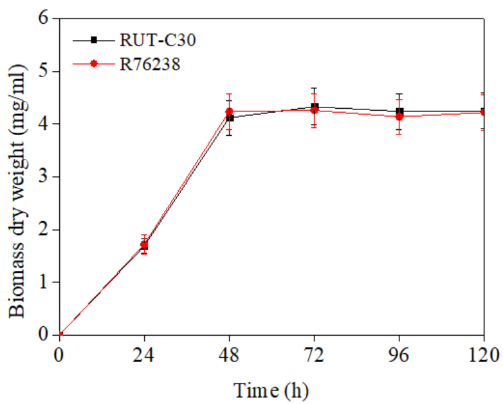

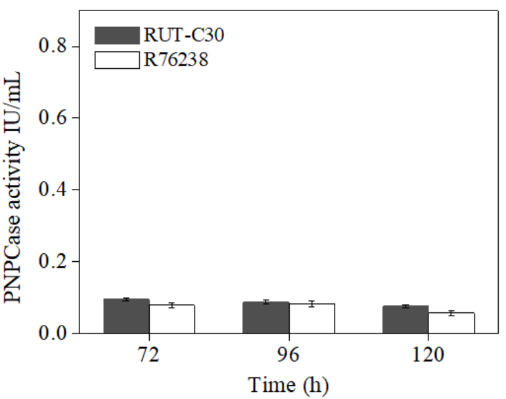
A B

**Fig. S8**


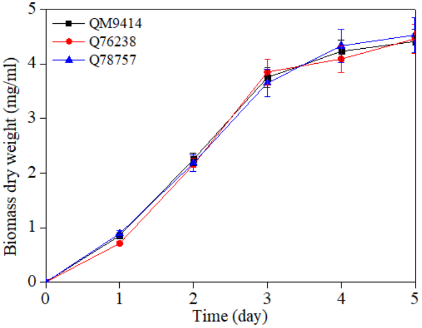

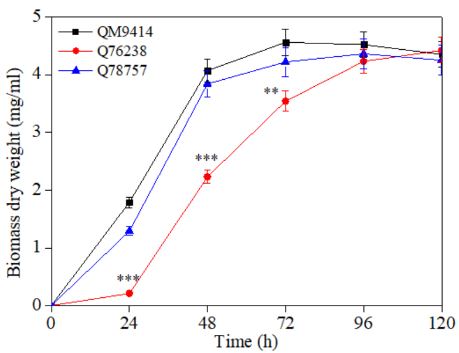

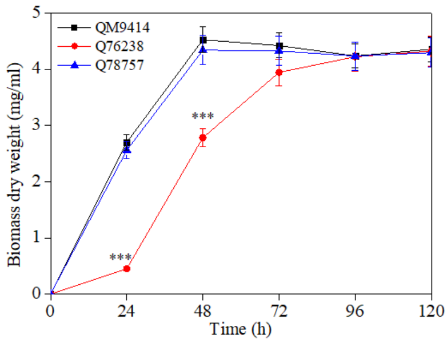
A B C

**Fig. S9**


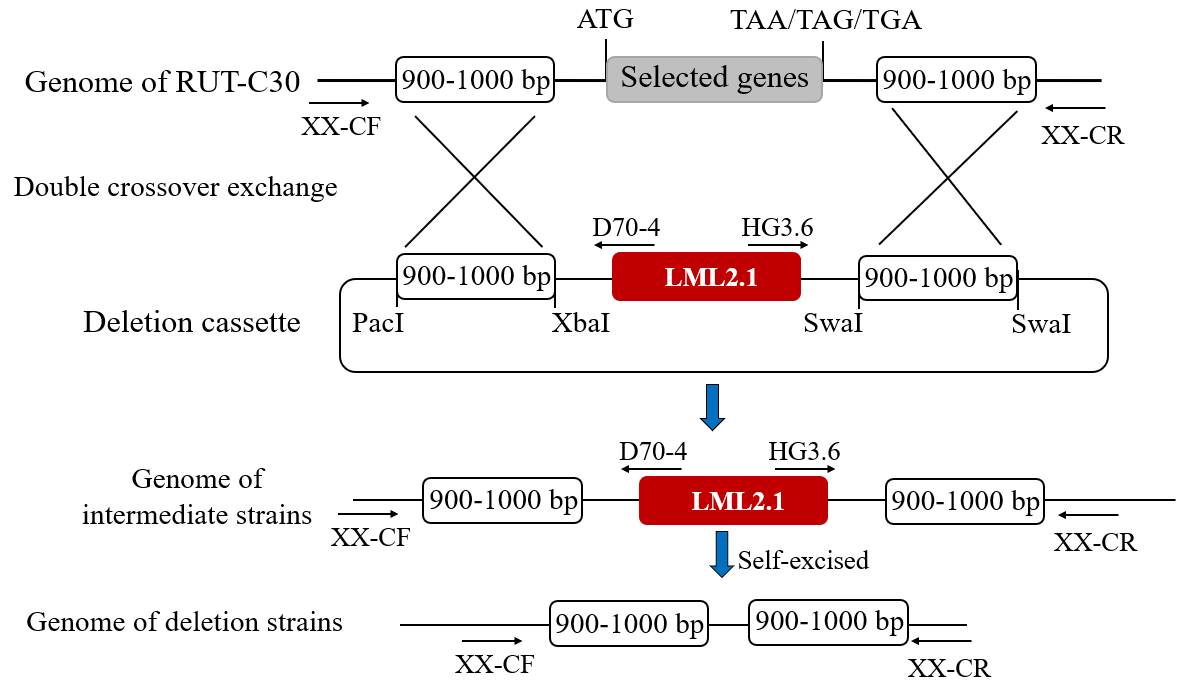


**Fig. S10**
